# Pancreatic Adenocarcinoma: Real World Evidence of Care Delivery in AccessHope Data

**DOI:** 10.3390/jpm13091377

**Published:** 2023-09-15

**Authors:** Afsaneh Barzi, Angela J. Kim, Crystal K. Liang, Howard West, D. Wong, Carol Wright, Nitya Nathwani, Catherine M. Vasko, Vincent Chung, Douglas A. Rubinson, Todd Sachs

**Affiliations:** 1 AccessHope, Duarte, CA 91010, USA; angela.kim@myaccesshope.org (A.J.K.); crystal.liang@myaccesshope.org (C.K.L.); jack.west@myaccesshope.org (H.W.); carol.wright@myaccesshope.org (C.W.); cathy.vasko@myaccesshope.org (C.M.V.); todd.sachs@myaccesshope.org (T.S.); 2 Department of Medical Oncology and Therapeutics Research, City of Hope, Duarte, CA 91010, USA; vchung@coh.org; 3 Department of Hematology and Hematopoietic Stem Cell Transplant, City of Hope, Duarte, CA 91011, USA; nitya.nathwani@myaccesshope.org; 4 Dana-Farber Cancer Institute, 450 Brookline Ave, Boston, MA 02215, USA; douglas_rubinson@dfci.harvard.edu

**Keywords:** pancreatic cancer, germline testing, comprehensive care, subspecialist care, care delivery

## Abstract

Background: Pancreatic adenocarcinoma is an aggressive disease and the delivery of comprehensive care to individuals with this cancer is critical to achieve appropriate outcomes. The identification of gaps in care delivery facilitates the design of interventions to optimize care delivery and improve outcomes in this population. Methods: AccessHope™ is a growing organization that connects oncology subspecialists with treating providers through contracts with self-insured employers. Data from 94 pancreatic adenocarcinoma cases (August 2019–December 2022) in the AccessHope dataset were used to describe gaps in care delivery. Results: In all but 6% of cases, the subspecialist provided guideline-concordant recommendations anticipated to improve outcomes. Gaps in care were more pronounced in patients with non-metastatic pancreatic cancer. There was a significant deficiency in germline testing regardless of the stage, with only 59% of cases having completed testing. Only 20% of cases were receiving palliative care or other allied support services. There was no difference in observed care gaps between patients receiving care in the community setting vs. those receiving care in the academic setting. Conclusions: There are significant gaps in the care delivered to patients with pancreatic adenocarcinoma. A concurrent subspecialist review has the opportunity to identify and address these gaps in a timely manner.

## 1. Introduction

Pancreatic ductal adenocarcinoma (herein referred to as pancreatic cancer) comprises more than 86% of all pancreatic malignancies [1]. In the United States, pancreatic cancer accounts for 3% of all cancer incidence and 7% of cancer mortality, making it the third leading cause of cancer death [2]. Approximately 45–55% of patients have metastatic disease at the time of presentation, and about 80–90% of patients have unresectable tumors, which are associated with poor prognosis [3].

Determining the correct stage of disease at diagnosis and ensuring timely and coordinated multidisciplinary care is critical to attaining optimal outcomes for patients with pancreatic cancer. In addition to oncologic treatment, other elements of management, including germline testing and the integration of supportive and palliative interventions, have an immense impact on the outcomes of patients with pancreatic cancer. In fact, comprehensive care is associated with improved survival in this population [4]. The degree to which comprehensive care is delivered across the U.S.’s pancreatic cancer population, however, is unknown. 

Patients treated at NCI-Designated Comprehensive Cancer Centers (NCI-CCCs) have improved cancer outcomes, including superior survival rates, compared with patients treated at other academic facilities or community practices. The improved outcomes are presumably due to high levels of specialization and experience, in turn due to the high volume of cases at NCI-CCCs [5,6]. The direct association between physician expertise and patient outcomes is well established in the surgical literature [7,8]. However, the precise impact of disease-specific expertise on patient outcomes in oncology is not well described [9]. While there are many factors that preclude all patients from being treated at NCI-CCCs, understanding the gaps in care of cancer patients will facilitate interventions to improve care, and ultimately outcomes, for all patients. The dissemination of disease-specific expertise via telehealth/remote means to a large, diverse network of community providers has been demonstrated to be a successful model for advancing care [10].

AccessHope™ is a rapidly expanding organization founded by City of Hope and supported by the growing cohort of NCI-CCCs across the U.S. to facilitate the remote delivery of cancer-specific subspecialist expertise to treating oncologists and their patients regardless of where they live, obviating the need for travel to a physical NCI-CCC site for consultation or ongoing care [11]. AccessHope serves a growing number of large self-insured employers and health plans across the country, facilitating collaboration with treating physicians to enhance care delivery to their patients. The process by which AccessHope identifies and reviews cancer cases, and then connects them with treating oncologists, has been previously described [11].

The quality of care of patients with pancreatic cancer has been a topic of interest for more than a decade, with most of the focus being on care delivered in the hospital setting and the quality of surgery [12]. However, most patients with pancreatic cancer have metastatic disease and are not candidates for surgery, and because of the multidisciplinary nature of management, even patients with non-metastatic pancreatic cancer receive most of their care in an outpatient setting. Therefore, it is important to understand care delivery patterns and gaps in care in the outpatient setting to allow the identification of areas needing improvement. 

Because AccessHope subspecialists review only outpatient cases, insights into practice patterns can be elucidated. By analyzing AccessHope data, one can identify and measure differences between care that is recommended by a subspecialist and that which is actually being delivered in the broader community. Therefore, using the AccessHope dataset as a benchmark, one is able to evaluate the quality and comprehensiveness of care in a geographically diverse population of patients with pancreatic cancer. 

Here, we present a descriptive analysis of care patterns and gaps in care observed in oncology community practices and non-NCI-CCC academic centers, compared with AccessHope NCI-CCC subspecialists. 

## 2. Materials and Methods

### 2.1. Case Identification

Candidate cases were identified via two parallel pathways: claims interrogation of contracted members (ICD-10 codes: C25.0–C25.9) for the prospective identification of patients OR patient request for a second opinion. The claims-based program, known as Accountable Precision Oncology (APO), runs as part of the health plan operations and without the need for direct request for review. The program of patient-requested review, known as Expert Advisory Review (EAR), is initiated by a contracted member or family member. 

### 2.2. Case Review

The details of case processing have been previously described [11]. Briefly, once a case has been identified, the AccessHope team obtains medical records and submits them for review to an NCI subspecialist in the AccessHope network, who then uses the records to render their recommendations. The specialist’s recommendations are then processed to ensure that they meet quality standards for comprehensiveness and clarity by an AccessHope team of nurse practitioners and oncologists. For APO, the final product is then shared with the health plan and the member’s treating physician(s). For EAR cases, the report is transmitted to the member’s treating physician and a copy of the report is shared with the member. 

### 2.3. Case Evaluation

An AccessHope clinical team routinely reviews all reports and adjudicates the projected impact of the recommendations on the quality of care delivered and the resulting outcome(s).

Quality of care is assessed in the context of the 6 domains of quality defined by AHRQ, including safety, efficacy, effectiveness, timeliness, patient centeredness, and equity [13,14]. The magnitude of these gaps is compiled in the AccessHope dashboard as the *Level of Concordance*. For this analysis, *Level of Concordance* was described in three main subgroups:

- Complete agreement with the care that is planned and delivered, labeled as “Agree”.
- Disagreement with the ongoing or planned cancer-directed care, with provision for recommendations for evidence-based care that confers safer and/or more effective and timely management, labeled as “Disagree”.
- Agreement with the overall cancer-directed therapy, yet with provision for recommendations for additional evaluation and/or interventions that would make care more efficient, patient-centered, and equitable. These cases are labeled as “Agree, with care enhancements”.

Additionally, the AccessHope clinical team assessed the impact of recommendations on member outcome(s). For this analysis, the improvements in outcomes were recorded in four main categories:

- Cancer-directed therapy;
- Improved member well-being;
- Benefit to family due to germline testing;
- Recommendations for relevant clinical trials.

The potential impact of recommendations on member outcome(s) can be assessed based on the data in the published literature. Any recommended changes in the current or future cancer-directed therapy with survival benefit are presumed to result in improved cancer-directed therapy. Recommendations for supportive and palliative care or suggestions for changes in cancer-directed therapy associated with a reduction in toxicity are presumed to improve member wellbeing. Recommendations for germline testing are presumed to have a beneficial impact on family members as well as the patient. For this analysis, we provide an overview of the gaps in care based on the categories listed above.

After a treating oncologist has received the AccessHope review, they can connect with an AccessHope oncologist to discuss the case and clarify any recommendations. A summary of the conversation with the treating oncologist is documented in the AceessHope dataset. This summary includes the receptiveness of the treating oncologist and the likelihood of implementation of AccessHope recommendations into their patient’s care. 

In addition to the data elements listed above, for this study, the patient’s location of care was documented based on whether the treating physician was affiliated with an academic or community practice.

The study was conducted according to the guidelines of the Declaration of Helsinki and approved by the City of Hope Institutional Review Board for analysis and publication.

## 3. Results

### 3.1. Case Characteristics

AccessHope reviewed 94 cases of pancreatic cancer between August 2019 and December 2022 (Supplemental Figure S1). The clinical characteristics of this cohort of patients are reported in Table 1. Cases were being managed across 30 states and the distribution between academic centers and community settings was 34% and 66%, respectively. Of these 94 cases, 86 (91%) were reviewed as part of the APO program, proactively identified through claims data, and the remainder of the cases were patient-initiated requests.

The cases were reviewed by specialists from several of the institutions in the AccessHope NCI-CCC network, which includes City of Hope, Dana-Farber Cancer Institute, Emory Healthcare and Winship Cancer Institute of Emory University, Fred Hutchinson Cancer Center, and Northwestern Medicine and the Robert H. Lurie Comprehensive Cancer Center of Northwestern University. 

### 3.2. Case Evaluations

**Figure 1:**
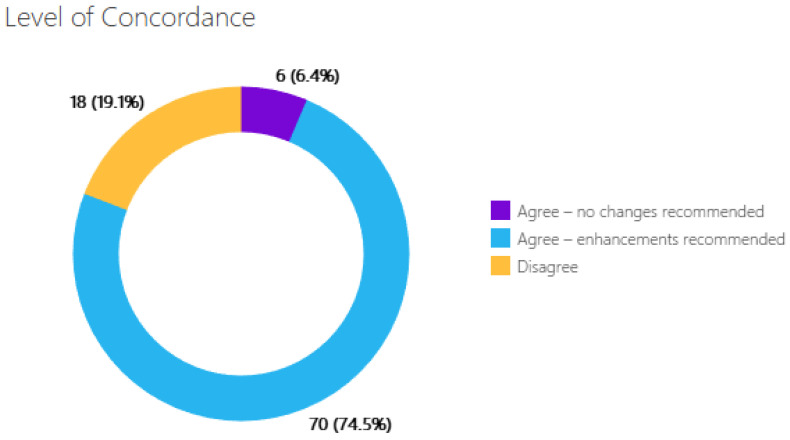
Distribution of Level of Concordance in the cohort. Only 6 patients were receiving comprehensive care; all others were recepients of care that had room for improvement. For each category, the number of cases is included in the graph and the percentages are in parentheses.

Analyzing quality of care using *Level of Concordance* demonstrated that only 6 of these 94 cases (6%) were classified as “agree”, with no additional recommendations (Figure 1). Nineteen percent of cases had a *Level of Concordance* of “disagree”, where the AccessHope expert was not aligned with the current approach to cancer-directed therapy and possibly other aspects of management. Examples of practices that led to discordance between the NCI-CCC recommendations and actual care included a lack of multidisciplinary evaluation for tumor resectability prior to initiating treatment; planning for and/or administering non-standard systemic therapies; and inadequate staging. In all other cases (75%), recommendations were “agree, with care enhancements”. The three most common recommendations across these cases were for germline testing, somatic testing (in cases with metastatic or locally advanced disease), and for the integration of supportive care services (all cases).

**Figure 2:**
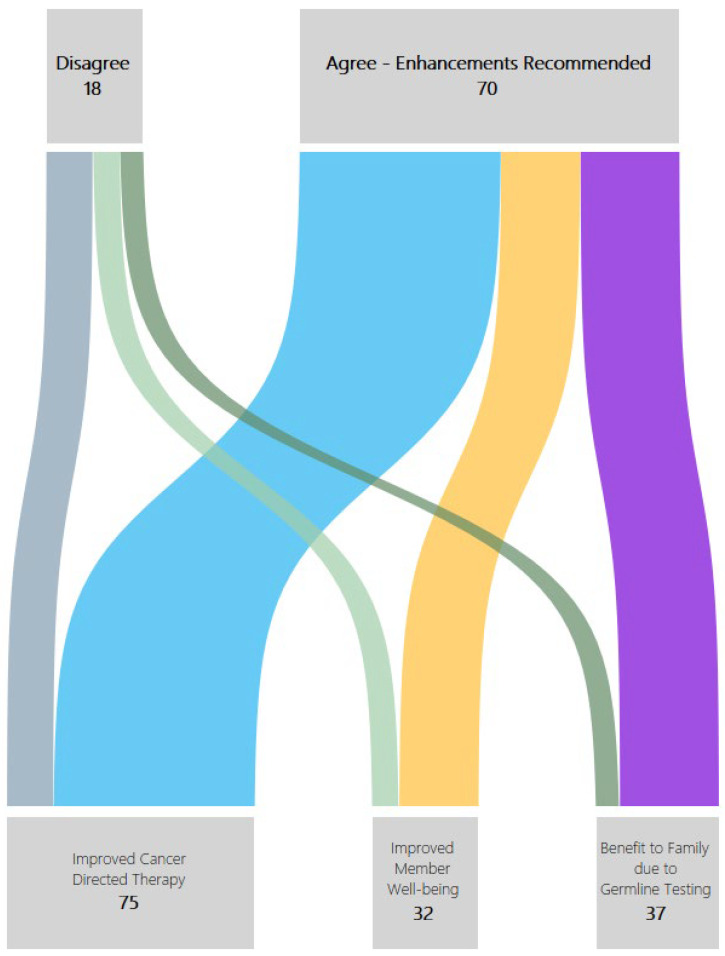
The relationship between Level of Concordance (a summation of gaps in care/quality of care) and impact on the individual’s outcomes. Gaps in care result in suboptimal cancer-directed treatment, member wellbeing, and a missed opportunity for cancer prevention in family members.

In 85% of cases, recommendations were made that, if adopted by the treating physician, had the potential to improve the current cancer-directed therapy and treatment planning. In 34% of cases, AccessHope recommendations correlated with an anticipated improvement in patient well-being. It is notable that each case can have more than one outcome. These potential improvements in patient outcomes were seen across all cases regardless of whether the Level of Concordance was “Agree with care enhancements” or “Disagree” (Figure 2). In addition, in 40% of cases, recommendations were made for one or more relevant clinical trials for which the patient would potentially be eligible.

### 3.3. Gaps in Care by Stage

The *Level of Concordance* “disagree” category was found to vary across the disease stage: 59 patients with metastatic disease (63%) accounted for 33% of the “disagree” cases, while 35 patients with localized and locally advanced disease (37%) accounted for 67% of the “disagree” cases. 

Evaluating the chemotherapy regimen provided to patients with metastatic disease, 81% of patients with metastatic disease received FOLFIRINOX in the front-line setting. Among patients with non-metastatic disease who received neoadjuvant-intent therapy, FOLFIRINOX was the predominant regimen (90%). 

### 3.4. Differences between Academia vs. Community

We evaluated the care received by patients treated at academic centers vs. those treated at community practices by looking at access to comprehensive care, including germline and somatic testing, as well as supportive and palliative care services (Table 2). Of the 94 pancreatic cancer cases reviewed, germline testing had been completed in 59%, with no difference in the completion of this test between academic and community physicians. Similarly, somatic testing had been completed in 69% of unresectable and metastatic cases with a comparable completion rate between academic and community settings. The majority of patients (80%) in the cases reviewed were not receiving supportive and palliative services, though there was a slightly higher rate of integration of these services at academic centers.

Direct communication between an AccessHope oncologist and the patient’s primary oncology provider was completed in 24 (25%) of pancreatic cancer cases. Treating physicians were receptive to the AccessHope recommendations and in 88% of the cases were open to implementing them (as long as there had been no change in the patient’s condition during the time between the review of the case and communication with the treating physician).

## 4. Discussion

Pancreatic cancer is one of the most lethal cancers, and the proper care and management of individuals with pancreatic cancer is pivotal in reducing their morbidity and mortality [15,16]. While intensive research into more effective therapeutics for pancreatic cancer and innovation in the space of earlier detection is important, there exists a gap between optimal evidence-based care and actual care that contributes directly to suboptimal outcomes for people with pancreatic cancer. In the AccessHope™ cohort of 94 patients, only 6% were receiving completely guideline-concordant comprehensive care, while the remaining 94% were receiving care that left room for improvement. This analysis highlights that a timely evaluation of patients for tumor resectability, the routine utilization of germline and somatic testing, and the integration of supportive care services remain major challenges in this broadly sampled U.S.-based population. 

It is notable that only 9% of patients in this cohort had self-initiated requests for a second opinion, the rest being prospectively identified through claims data. While a second opinion is highly encouraged in oncology [17], patients with pancreatic cancer, who often have a heavy symptom burden and complex treatment plans, may not be able to seek a second opinion. Importantly, most patients seeking a second opinion are looking to verify the appropriateness of their cancer-directed regimen [18], unaware of the other integral aspects of care, such as germline testing or the integration of ancillary support services, that would likely improve their outcomes. When we looked at how the Level of Concordance correlated with anticipated clinical and humanistic impact on the individual level, we found that many patients were receiving treatment that was broadly appropriate (for example, FOLFIRINOX). Yet, recommendations by the AccessHope specialist for personalized modifications to the care plan—such as optimization of the treatment dose and schedule, and/or pain management—translate to large gains at the individual level, such as by improving member well-being (Figure 2). 

Our case analysis demonstrates that germline testing was not considered or offered to a significant portion of the patients (41%) for whom it would be appropriate according to national guidelines. Moreover, several cases had testing for BRCA mutation only, rather than universal panel testing. The barriers to germline testing and genetic counseling are well documented in patients with cancer, and pancreatic cancer is no exception [19,20]. We observed no difference in the completion of germline testing between academic and community sites. Because pancreatic cancer is a lethal disease, identifying those patients with a germline mutation provides an opportunity for personalized cancer prevention in first-degree relatives [21]. Therefore, the societal effect of inadequate germline testing, including universal germline testing, extends beyond the care of the patient in question and deserves special attention [22,23]. Additionally, given that patients with BRCA1 or BRCA2 mutations are candidates for olaparib, a lack of germline testing limits the delivery of effective, FDA-approved precision medicine to those who would benefit from it.

Despite recommendations for the universal use of supportive and palliative care for patients with pancreatic cancer [24], only 19% of patients in this cohort had documentation of referral to palliative and supportive care services. While it is possible that a lack of documentation may explain, at least in part, the low utilization of supportive resources observed in this study, the large difference between the use of other ancillary services like germline testing and the use of supportive services suggests a need for measures that promote awareness and a more consistent implementation of supportive services in the oncologic care of patients with pancreatic cancer.

A discordance between AccessHope recommendations and actual care was more prevalent in cases where patients had non-metastatic disease; for these patients, multidisciplinary care is crucial, and the discordance seen in this study and others suggests that fragmented care may be a significant contributor to suboptimal care [25,26]. While the identification of patients with metastatic disease is straightforward, delineating which patients have borderline resectable or unresectable tumors requires the skilled use of imaging modalities and a careful review of the findings in a multidisciplinary and cohesive manner [27,28,29]. Our observation suggests that patients with non-metastatic disease stand a significant chance of receiving suboptimal treatment and follow-up due to the ambiguity of the stage at diagnosis. It is critical to point out that the use of chemotherapy regimens observed in our cohort was concordant with NCCN guidelines (i.e., the majority of patients received FOLFIRINOX). However, there were significant gaps in care patterns, ranging from early surgery for unresectable cancer without appropriate assessment by a multidisciplinary team to starting induction chemotherapy without input from a surgical subspecialist. In light of the recent publication of trials suggesting potential detrimental effects of neoadjuvant therapy in patients with resectable pancreatic cancers, the practice of starting with chemotherapy prior to the delineation of resectability should be aborted [30,31]. Therefore, strategies to facilitate timely input by an expert provider or team of providers may optimize care delivery in this population regardless of where care is being delivered [32].

We noted a high rate of FOLFIRINOX usage in our cohort of cases, with a wide variation in dosing. While it is encouraging to see the frequent adoption of this highly-active regimen in pancreatic cancer [33,34], we observed no clear practice patterns to reduce the risk of oxaliplatin-induced neurotoxicity associated with FOLFIRINOX. Furthermore, the use of growth factors for the primary prevention of neutropenia was sparse. These findings suggest that while appropriate anti-cancer therapy may be utilized, proactive supportive interventions to prevent or mitigate treatment-related toxicity can be improved.

Our data revealed that academic and community sites demonstrated similar opportunities to improve the comprehensiveness of care of patients with pancreatic cancer. This suggests that access to specialist services (like genetic counseling and germline testing or supportive care) may not be the prevailing barrier to integrating these services into the care plan. One intervention that may lead to improved comprehensive care across all outpatient settings is proactive case review and peer-to-peer communication, such as using the AccessHope service, which may have tremendous benefits for patients and their families.

The strength of this case series is that it provides insight into multiple aspects of care—in addition to cancer-directed therapy—of a diverse sampling of patients with pancreatic cancer across the U.S. Additionally, findings from this cohort more closely reflect the reality of current practice patterns rather than a retrospective cohort, who may have received care at a time when paradigms of care were different. It is critical to mention that in this cohort, all patients had private insurance, indicating that the quality or comprehensiveness of their care cannot be attributed to a lack of coverage of medical services deemed to be optimal or standard-of-care.

As for limitations, this case cohort represents a small and heterogenous population of individuals with pancreatic cancer covered through employer-funded insurance, and thus the results may not be generalizable. Furthermore, due to the nature of AccessHope case reviews being reliant on available clinical documentation, it is possible that some elements of care that were actualized were not captured in this study and are thus underestimated. However, relying on clinical documentation to measure quality of care in oncology practice is the norm for larger nationwide programs such as the American Society of Clinical Oncology’s Quality Oncology Practice Initiative (QOPI^®^) [35]. 

Finally, it is important to note that AccessHope does not have a longitudinal follow-up of these cases to assess the impact of reviews on management. We relied on the testimony of treating physicians for the implementation of recommendations. Due to this limitation, AccessHope has begun the process of verification of the implementation of recommendations through a number of mechanisms, including the use of claims as well as medical records. Findings of these efforts will be included in future analyses. 

## 5. Conclusions

This expert subspecialist review of contemporary cases of pancreatic cancer through the AccessHope program revealed significant gaps in the care of pancreatic cancer patients in the U.S. These gaps include the inadequate use of germline testing and palliative and supportive care services for all patients with this devastating disease, regardless of where care was delivered (i.e, academic center vs. community). Moreover, this work highlights notable deficiencies in the care of patients with non-metastatic pancreatic cancer; these individuals appear to be at particular risk of receiving suboptimal care, in large part due to a seeming lack of coordination of the multidisciplinary components of care required at diagnosis.

The recognition of these deficiencies provides a basis for designing quality improvement projects at the practice level. Additionally, access to interventions, such as the AccessHope program, will assist with care optimization and facilitate the delivery of high-quality care to each and every patient with cancer.

## Figures and Tables

**Table 1:** Clinical characteristics.

Clinical Case Characteristics of the Cohort	
Age, Median (range)	58 years (38–81)
Gender, Male (%)	55%
Stage, Metastatic (%)	63%
Treating Physician, Academic (%)	34%

Category stage refers to stage at the time of case review with AccessHope.

**Table 2:** Gaps of care in academic and community settings. Somatic testing was evaluated only in cases of unresectable and metastatic disease.

	Germline Testing		Somatic Testing		Supportive Care Integration	
	Academic	Community	Academic	Community	Academic	Community
**Missing**	13 (41%)	25 (40%)	8 (39%)	12 (28%)	24 (75%)	52 (84%)
**Completed**	19 (59%)	37 (60%)	14 (64%)	31 (72%)	8 (25%)	10 (16%)

## Data Availability

Not applicable.

## Supplementary Materials

The following are available online at https://www.mdpi.com/article/10.3390/jpm13091377/s1. Figure S1. Volume of cases per year over the study period.
