# Supplementary figures and images for: Pancreatic Adenocarcinoma: Real World Evidence of Care Delivery in AccessHope Data
Source: J Pers Med. 2023 Sep 15;13(9):1377. doi: 10.3390/jpm13091377 (PMC10532778; doi:10.3390/jpm13091377)

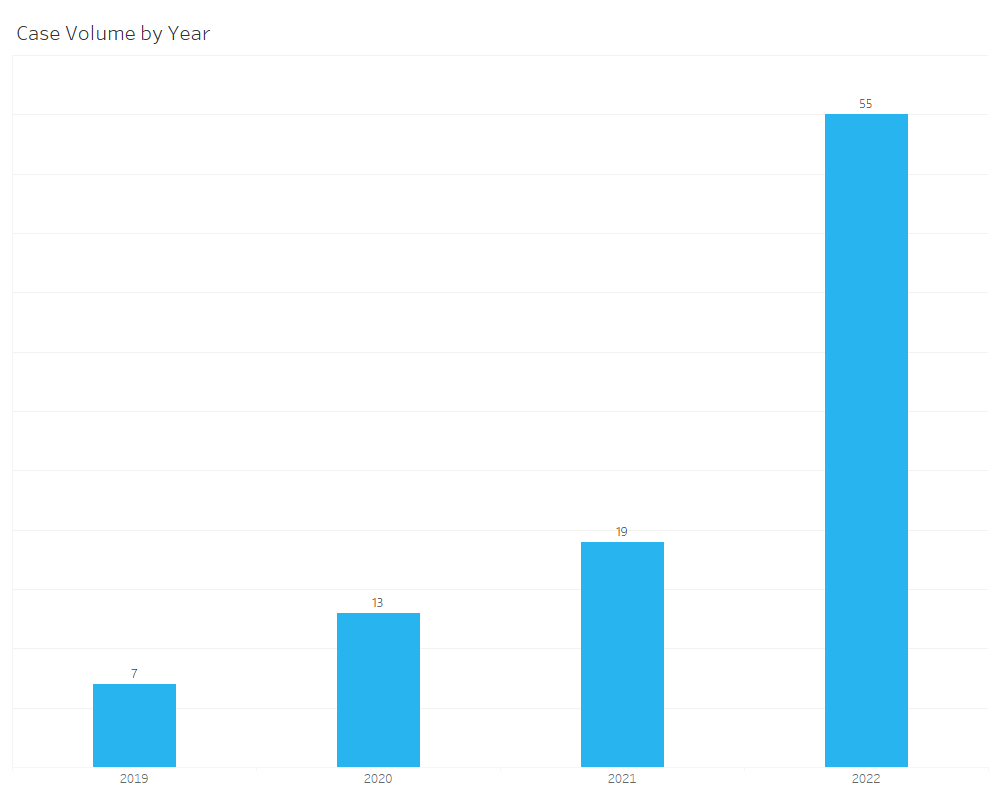

Supplement: Supplementary file 1 [file jpm-13-01377-s001.zip › jpm-2534430-supplementary.png]
